# How well did our students match? A peer-validated quantitative assessment of medical school match success: the match quality score

**DOI:** 10.1080/10872981.2019.1681068

**Published:** 2019-10-29

**Authors:** Chayan Chakraborti, Jason E. Crowther, Zachary A. Koretz, Marc J. Kahn

**Affiliations:** a Department of Medicine, Tulane University School of Medicine, New Orleans, LA, USA; b Resident in Surgery, Harbor-UCLA Medical Center, Los Angeles, CA, USA; c Resident in Internal Medicine, Tulane University School of Medicine; d Office of Admissions and Student Affairs, Tulane University School of Medicine, New Orleans, LA, USA

**Keywords:** Undergraduate medical education, residency match, career advising, NRMP, matching

## Abstract

**Background**: Assessment of an individual medical school’s performance in the match is an important outcome of the educational program. Unfortunately, student rank lists are not public. A method to objectively gauge the quality of an institution’s match regardless of student preference has not been described in the literature.

**Objective**: This manuscript serves to determine the relative weights of included variables and derive a statistically valid Match Quality Score (MQS).

**Design**: Between 2016 and 2018, student affairs experts derived from a national cohort validated the MQS by scoring factitious mini-match lists that covered three variables: student’s *Match Status*, specialty *Competitiveness*, and residency program *Reputation*.

**Results**: Of the variables assessed, only *Match Status* and *Competitiveness* were found to be significant. We derived the resulting coefficients for the Match Quality Score (MQS) as: [3.74A (# students successfully matched) + 2.34B (# students matching into their initial specialty in the SOAP process) + 1.77C (# students who secured a SOAP position in another specialty) + 0.26D (# students matching into a specialty where there are more applicants than spots)]/Total # students.

**Conclusions**: The MQS is a potentially useful educational outcome measurement for US medical schools and may be considered as an outcome measure for continuous quality improvement to tailor future institutional changes to training, mentoring, and student-advising programs.

## Background

The quality of a medical school’s academic enterprise may be assessed by several metrics: scores achieved on standardized exams (USMLE, NBME), accreditation status, and results of the Graduate Questionnaire (GQ). Results of the residency match are another indication of the effectiveness of the medical education program [1]. While analysis of match results can be found in the literature [2–4], these are primarily from the perspective of graduate medical education programs (residencies), and quality estimations are limited to the number of programs/positions that did not fill, or from a basic comparison of overall applicant number to positions available [2–7]. Match results from undergraduate medical institutions, when reported at all, are comprised of qualitative assessments of the match performance that are subjective and prone to bias, making year-to-year comparisons between matches difficult, if not impossible. An earlier study sought to assess the quality of match success by surveying program directors at the home intuition and was able to provide a subjective description their students’ placement at various residency locations [8]. However, such a study is subjective and less useful for continuous quality improvement. To truly improve the quality of a medical school’s match outcomes, a quantitative method should be developed to ‘score’ the matches from year to year. With such a scoring process, schools could compare annual match performance to assess the impact of changes in curriculum or advising.

## Objective

For over a decade at Tulane University School of Medicine, a quantitative scoring system has been used to rate successful matches in the National Residency Matching Program (NRMP), Military Match (MM), Ophthalmology Match (SF), American Urologic Association Match (AUA), or Supplemental Offer and Acceptance Program (SOAP). This scoring system was initially based on a weighting of match outcome variables that were assumed in a non-scientific fashion (numbers matched, competitiveness of residencies matched into, etc.). In 2016, we sought to validate the scoring system by using a panel of national experts to score artificial match lists generated to determine the relative weights of included variables using linear/logistic regression and derive a final Match Quality Score (MQS). The MQS was intended to utilize easily obtainable, institution-level data. In this validation pilot, we did not include elements such as student’s match perspective, institutional objectives, or quality of advising. Rather, we only used readily available outcome variables that would be easily accessible to any medical school.

## Methods

For the study design, we created 24 factitious mini-match lists composed of 10 factitious students, each covering the range of three independent variables: *Matching Status, Competitiveness*, and *Reputation*. For *Matching Status* there were four possible outcomes: matching in the initial match (either NRMP, MM, SF, or AUA), matching by SOAP into the same specialty, matching by SOAP into different specialty, and failing to match. *Competitiveness* was defined as the ratio of US senior applicants who successfully matched into their preferred specialty to the number of total positions available in the specialty. The 18 included specialties in the NRMP match plus specialties from the other matches (urology, ophthalmology) were grouped based upon their applicants/available positions ratios, with three possible outcomes: matching into a highly competitive specialty, matching into a moderately competitive specialty, and matching into a less competitive specialty (see Table 1) [9–11]. *Reputation* of the institution, adapted from methods described in other studies [8,12], was binary: whether or not the matched program was among the top 10 in terms of NIH funding. Each factitious mini-match list fulfilled one condition: *Matching Status* (four possible outcomes) x *Competitiveness* (three possible outcomes) × *Reputation* (two possible outcomes).

Twenty-four artificial match lists for 10 fictitious students were randomly generated. Out of these 10 fictitious students, two student’s match results were part of the experimental manipulation for each of the artificial match lists. Each of these two experimental students match results varied by one of the study variables: *Matching Status* (four possible conditions: Matched, Unmatched-SOAP in same specialty, Unmatched, SOAP in different specialty, Unmatched), *Competitiveness* of match specialty (three conditions: High, Medium, Low), or program *Reputation* (2 conditions: Top 10 NIH-funded, not-Top-10). The specific order that the two fictitious students appeared in the artificial match list was randomized (see Table 2).

**Table 1.** Competitiveness of different medical specialties used in the study according to the ratio of US senior applicants who successfully matched into their preferred specialty to the number of total positions available in the specialty.

High	Medium	Low
Orthopedic surgery	General Surgery	Psychiatry
Otorhinolaryngology	Obstetrics/gynecology	Neurology
Neurosurgery	Emergency Medicine	Physical Medicine
Dermatology	Pediatrics	Internal Medicine
Ophthalmology	Anesthesiology	Family Medicine
Plastic Surgery	Diagnostic radiology	Pathology
Urology		
Radiation Oncology

**Table 2.** Description of 24 artificial match lists delivered in a randomized fashion to be scored by survey respondents.

Fictitious Match	Reputation^a^	Competitiveness^b^	Matched^c^
Set 1	0	0	0
Set 2	0	0	1
Set 3	0	0	2
Set 4	0	0	3
Set 5	0	1	0
Set 6	0	1	1
Set 7	0	1	2
Set 8	0	1	3
Set 9	0	2	0
Set 10	0	2	1
Set 11	0	2	2
Set 12	0	2	3
Set 13	1	0	0
Set 14	1	0	1
Set 15	1	0	2
Set 16	1	0	3
Set 17	1	1	0
Set 18	1	1	1
Set 19	1	1	2
Set 20	1	1	3
Set 21	1	2	0
Set 22	1	2	1
Set 23	1	2	2
Set 24	1	2	3

^a^ Reputation: 0 = not Top-10 NIH funding; 1 = Top-10 NIH funding

^b^ Competitiveness: 0 = Low; 1 = Medium; 2 = High

^c^ Matching Status: 0 = failing to match; 1 = SOAP into different specialty; 2 = SOAP into same specialty; 3 = successful initial match

Student affairs experts were identified by membership in the Group on Student Affairs (GSA) within the Association for American Medical Colleges (AAMC). Members of the GSA hold leadership positions in US medical schools and have expertise relating to student career planning, student academic performance, and the residency matching processes. Between 2016 and 2018, student affairs expert participants were recruited via email through the GSA, and were supplied with a link to an online survey (Qualtrics®, Provo, UT, USA). The survey provided a tutorial which was followed by the 24 experimental lists, with each student affairs participant seeing the lists in a random order. Student affairs experts ranked each list on a 1 (poorest match) to 7 (best match) scale. Finally, student affairs experts answered questions about the three variables, as well as providing personal demographics including age, gender, and role in student affairs.

A repeated-measures analysis of variance (ANOVA) was performed to identify significant variables and post-hoc analysis was used to compare levels of different variables. We collapsed variables or levels which were not significantly different in subsequent analysis, and a Bonferroni correction was conducted on all pair-wise comparisons of the levels of main effects. All statistical analyses were performed using SPSS for Macintosh, Version 22.0 (IBM Corp. Armonk, NY). As the match lists and students were fictitious, and the survey responses were anonymous, this study was determined to be exempt per the Tulane Institutional Review Board.

## Results

Out of 141 possible respondents, 27 expert participants (16 women) completed the study in its entirety (response rate, 19.1%). Eighteen of the participants were student affairs deans at medical schools, three were student affairs faculty members, three were student affairs staff, and three elected not to disclose their specific role in student affairs.

Results from the ANOVA analysis indicated that the main effects of *Matching Status* (F(3,78) = 113.05, MS_error_ = 2.28, p < 0.001) and *Competitiveness* (F(2, 52) = 9.66, MS_error_ = 0.37, p < 0.001) were both significant, while the main effect of *Reputation* (F(1, 26) = 0.713, MS_error_ = 0.42, p = 0.41) was not. There were no interactions among any of the variables that were statistically significant or approached statistical significance (all p > 0.30).

Post-hoc analysis demonstrated that all 10 pair-wise comparisons of main effects were significant (pair-wise alpha = 0.005), except for the comparison between the institutional NIH rankings and between the specialty conditions of low-competitiveness to medium-competitiveness (see Table 3). As such, these non-significant levels were treated as equivalent in subsequent analysis.

**Table 3.** Weight estimates based upon repeated-measures ANOVA model.

Variable	Level	Weight	95% confidence interval	p
Matching	Matching^a^	3.74	(3.21, 4.26)	<0.001
	SOAP match, same specialty^a^	2.34	(1.91, 2.78)	<0.001
	SOAP match, different specialty^a^	1.77	(1.41, 2.13)	<0.001
Specialty	Competitiveness High^b^	0.26	(0.11, 0.41)	0.002
Institution	Top 10 institution^c^	0.05	(−0.07, 0.18)	0.406

^a^ Compared to unmatched

^b^ Compared to medium/low competitiveness

^c^ Compared to institutions not in top 10 of NIH funding

**Figure 1.**
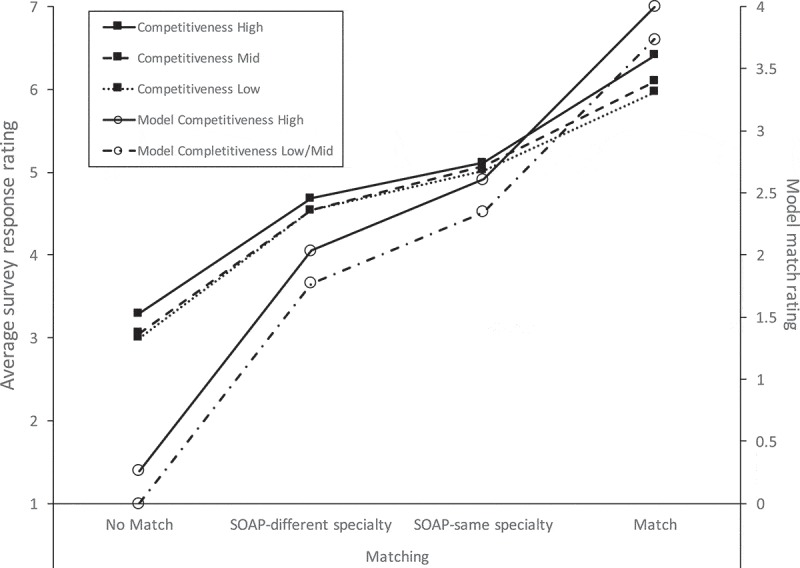
Average survey responses (left axis) and corresponding model results (right axis) by the matching status of factitious medical students. Ratings are from 27 student affairs experts employed by US medical colleges, 2016.

From the above results, we sought to derive an equation that would generate a matching score for individual applicants, restricting the range of possible scores from 0.0 to 4.0 analogous to a grade-point average (GPA), for ease of interpretation. To accomplish this, we first calculated the means for the individual conditions of *Matching Status* x *Competitiveness*, with medium- and low-competitiveness averaged (as these were not significantly different from one another in the post-hoc analysis). We performed a linear transformation of the means from a 1 to 7 scale onto a 0.0 to 4.0 scale, setting the lowest mean as 0.0 (failing to match into a medium/low-competitive specialty) and the highest as 4.0 (matching on the initial match into a highly competitive specialty). This process is graphically displayed in Figure 1, in which empirically derived means on the 7-point scale for the significant main effects are shown on the left vertical axis and the ANOVA model results, with linearly transformed means, are shown on the right vertical axis. Using this process, we calculated the weights for the different significant variables and levels (see Table 3). The resulting validated calculation for match performance, derived from our data, is:

$$MQS\;=\;\left(3.74A\;+\;2.34B\;+\;1.77C\;\;\;\;\;\;\;\;\;\;\;\;\;\;\;\;\;\;\;+\;0.26D\right)\;/\;Total\;\#\;of\;students\;in\;class\;match$$

Where A = number of students successfully matched; B = number of students matched by SOAP in *preferred specialty*; C = number of students matched by SOAP into *alternative specialty*; and D = number of students matching into a more competitive specialty (see Table 1).

## Discussion

A mandate of a medical school is to prepare students in such a way as to be successful in securing a residency position. Institutions achieve this through complex interactions between faculty, advisors, and students to establish learning environments sufficient to foster the requisite student knowledge, skills, attitudes, and behaviors, as well as providing an advisory system to guide students to appropriate specialties and programs. A medical school’s performance in the match is, therefore, one metric by which that institution may be judged and can be used to assess the effectiveness of a school’s educational programs over time. While physicians are by nature quantitative [13], in one 2001 survey-driven study of internal medicine residency program directors, the determination of quality of the match was a subjective assessment of ‘matched much better/somewhat better/same/somewhat worse/much worse than previous [years]’[14].

Our study differs from another prior study [8] in the use of hypothetical match lists to determine the impact of variables on the interpretation of match success. In addition, our survey methodology included assessments made by student affairs experts from multiple institutions as opposed to a single center. Finally, we examined several variables in an effort to derive a scoring system to estimate the quality of an institution’s match for a given year. Our study addresses the need for a quantitative objective assessment of an institution’s match outcome in a simple equation that can be used (and developed further) by schools over time.

As mentioned previously, the office of student affairs at Tulane previously used an algorithm to assess match performance based upon the variables included in the current study. While this study largely supported the previously used algorithm as a method for assessing match performance, we observed some expected empirical differences, such as the changes in the weights of the variables. The analysis also revealed some unexpected findings; notably, specialty competitiveness was slightly different from what was expected. Student affairs experts rated the quality of match higher if the students were trying to match into more competitive specialties, regardless of the fictional student’s match success into that specialty (see Figure 1). Additionally, there was no apparent effect of the reputation of the institution into which a student matched; though NIH funding may not be the optimal proxy for reputation. However, the difficulties of determining a program’s ‘reputation’ are consistent with findings from an earlier study [12].

Our study variables also did not incorporate the stated mission of a medical school. It may be that some residency programs’ alignment with a specific mission may overshadow that programs perceived external reputation. For example, if a student’s preference for primary care coincides with a residency program’s stated mission (e.g., primary care), this fit may be deemed more important than an overall national reputation. A student’s preference for a particular geographic area may also supersede reputation. In this case, the MQS equation could be modified to include an additional terms for ‘alignment with stated mission’ or ‘preferred geographic locale’. Quantifying these additional variables would need further investigation and validation as well as a way to obtain these preferences from individual students.

Individual medical student match success is a multifactorial process that includes academic performance, perceived quality of the medical school, suitability for a given program as judged by interviews, and student preference [15]. Importantly, our study focused on the quantitative assessment of a school’s match. Thus, this brief report has several key limitations; foremost is that it does not address factors that would make an individual student feel that their match was successful. Such a study focusing on students rather than the school would have to consider many local variable such as the impact of specific institutional missions and individual applicants’ perspectives. Additionally, to complete such a study, we would have to know how individual students ranked programs on their match rank lists. This would have required breaking the promised anonymity of individual rank lists, which is not possible according to rules established by the National Residency Matching Program [16]. Nevertheless, should institutions choose to voluntarily obtain rank-list information from their students, we suggest that the MQS may be used in conjunction with individual-level data to inform institutions more fully on their educational program and advising efforts.

With respect to advising students on residencies, the notion of ‘fit’ often becomes more prominent than factors such as a residency program’s reputation or competitiveness. The position that a residency program earns on an individual student rank list may have more to do with goodness-of-fit of the programs to which a given student applied and the knowledge of the advisors at their medical school when recommending programs. We suggest that the inclusion of an institution-level assessment such as the MQS in combination with ‘ground-level’ data from students and advisors may be more formative than either alone.

Finally, our validation set only included a limited number of variables such as numbers of students matching, successful matching in SOAP, matching into a competitive specialty, and the perceived quality of the matched program. Additional variables to assess the match quality of a program may be important but were not part of our initial study. Although the number of experts forming the validation cohort was small, the results were robust, and the participants were selected from a diverse group of individuals who are considered experts in the field of student affairs.

We can draw two primary implications of the present study. First, by using easily obtainable, institution-level data, the MQS provides a quantitative appraisal for a school’s match over time. Second, sub-analysis using the MQS stratified by a school’s constituent departments (i.e., the medicine department MQS, the psychiatry department MQS, etc.) may help continuous quality improvement efforts by assessing and refining the quality of the advising within a particular department. At our institution, we have been able to use the departmental MQS as a way to herald which departments may have a need for additional faculty development related to student residency advising or early career guidance.

## Conclusion

We believe that the MQS provides an initial step in developing a peer-validated method to quantify a medical school’s match performance, providing an overall assessment of an institution’s educational program effectiveness, while not requiring an institution to break student confidentiality. Validation efforts will need to evolve continuously as more institutions adopt the MQS and identify additional variables to be included in the MQS equation over time. We suggest that the MQS is a useful educational outcome measurement for medical schools that can be easily calculated annually, can be trended over time, and can be considered as an outcome measure for continuous quality improvement to tailor future institutional changes to training, mentoring, and student-advising programs.
